# Toxic effect of titanium dioxide nanoparticles on corneas *in vitro* and *in vivo*

**DOI:** 10.18632/aging.202412

**Published:** 2021-02-01

**Authors:** Junxing Yang, Jianliang Liu, Ping Wang, Juanjuan Sun, Xiaohui Lv, Yumei Diao

**Affiliations:** 1 Department of Ophthalmology, Weifang Medical University, Weifang 261042, Shandong Province, China

**Keywords:** titanium dioxide nanoparticles, corneal endothelial cells, oxidative stress, Nrf2

## Abstract

Titanium dioxide nanoparticles (TiO_2_ NPs) are widely used in a variety of areas. However, TiO_2_ NPs possess cytotoxicity which involves oxidative stress. Nuclear factor erythroid 2-related factor 2 (Nrf2) is a key molecule preventing cells from oxidative stress damage. In the current study, we explored the effect of Nrf2 signaling pathway in TiO_2_ NPs-induced corneal endothelial cell injury. Firstly, we found TiO_2_ NPs inhibited proliferation and damaged morphology and mitochondria of mouse primary corneal endothelial cells. Moreover, TiO_2_ NPs-induced oxidative damage of mouse primary corneal endothelial cells was inhibited by antioxidant NAC by evaluating production of reactive oxygen species (ROS), malondialdehyde (MDA), and activities of superoxide dismutase (SOD) and glutathione peroxidase (GSH-Px). Next, flow cytometry analysis showed TiO_2_ NPs promoted apoptosis and cell cycle G2/M phase arrest of mouse primary corneal endothelial cells. Further investigation suggested that Nrf2 signaling pathway activation and the downregulation of ZO-1, β-catenin and Na-K-ATPase were involved in TiO_2_ NPs-induced mouse primary corneal endothelial cell injury. Our research highlighted the toxic effect of TiO_2_ NPs on corneas *in vitro* and *in vivo*, providing an alternative insight into TiO_2_ NPs-induced corneal endothelial cell injury.

## INTRODUCTION

Nanomaterials are applied in food, medicine, cosmetics and other fields as the rapid development of nanotechnology [1, 2]. Recently, considerations have been given to the multifunction of titanium dioxide nanoparticles (TiO_2_ NPs), a typical and widely applicable nanomaterial. However, it could not be ignored that the cytotoxic effect, immunotoxicity and inflammatory response of TiO_2_ NPs exist when cells and organisms are exposed to TiO_2_ NPs [3–5]. The toxic effect and the underlying mechanism are largely unclear.

In general, TiO_2_ NPs could enter into the body through multiple approaches, like injection, inhalation and penetration [6], and cause damage of skin [7], respiratory system [8], brain [9], liver and other organs [10]. It should be concerned that NPs have been used as a topical ocular drug delivery system, the potential damage caused by NPs cannot be ignored [11]. Notably, the NPs lead to a series of ophthalmic diseases such as corneal disorders [12] and retinal diseases [13], because of the direct contact between eyes and the NPs [14]. Accumulating evidence has reported that other types of NPs are also involved in the eye injury. Lee et al demonstrate that zinc oxide NPs-induced upregulation of Bax and heme oxygenase participates in the rabbit cornea cell injury, while silver NPs, TiO_2_ NPs and silica oxide NPs have no effect on the expression of the two proteins [15]. A recent study shows that silver NPs destroy mitochondrial structure to aggravates cornea cell damage [16]. Therefore, the mechanisms of NPs-induced cytotoxicity vary according to the type of NPs. It has been demonstrated that TiO_2_ NPs-induced oxidative stress contributes to the pathogenesis of damage of cells and organs [17]. The increased generation of reactive oxygen species (ROS) and the decreased antioxidant effect are major reasons to cause oxidative stress. When organisms are exposed to TiO_2_ NPs, the excessive ROS induce by TiO_2_ NPs results in the inactivation of cell membrane proteins and damage of mitochondria and DNA, so as to generate toxic effects [17]. A recent research reported that three kinds of metal oxide NPs (TiO_2_ NPs, zinc oxide NPs and polyvinylpyrrolidone capped zinc oxide NPs) were tested to select a preferable vehicle to be used in the treatment of glaucoma, and TiO_2_ NPs were not suitable for further application because of the cytotoxicity of TiO_2_ NPs [18]. Another study reported that TiO_2_ NPs facilitated production of ROS and jeopardized the intracellular calcium homeostasis to inhibit proliferation of lens epithelial cells [19]. However, little is known about the role of TiO_2_ NPs in corneal endothelial cells and its underlying mechanism.

In the present study, we investigated the toxic effect of TiO_2_ NPs on corneal endothelial cells by using *in vitro* assays and *in vivo* experiments, highlighting a critical role of nuclear factor erythroid 2-related factor 2 (Nrf2)/antioxidant response element (ARE) signaling pathway in the pathogenesis of TiO_2_ NPs-induced damage of corneal endothelial cells.

## RESULTS

### TiO_2_ NPs boosted cell damage of corneal endothelial cells

**Figure 1:**
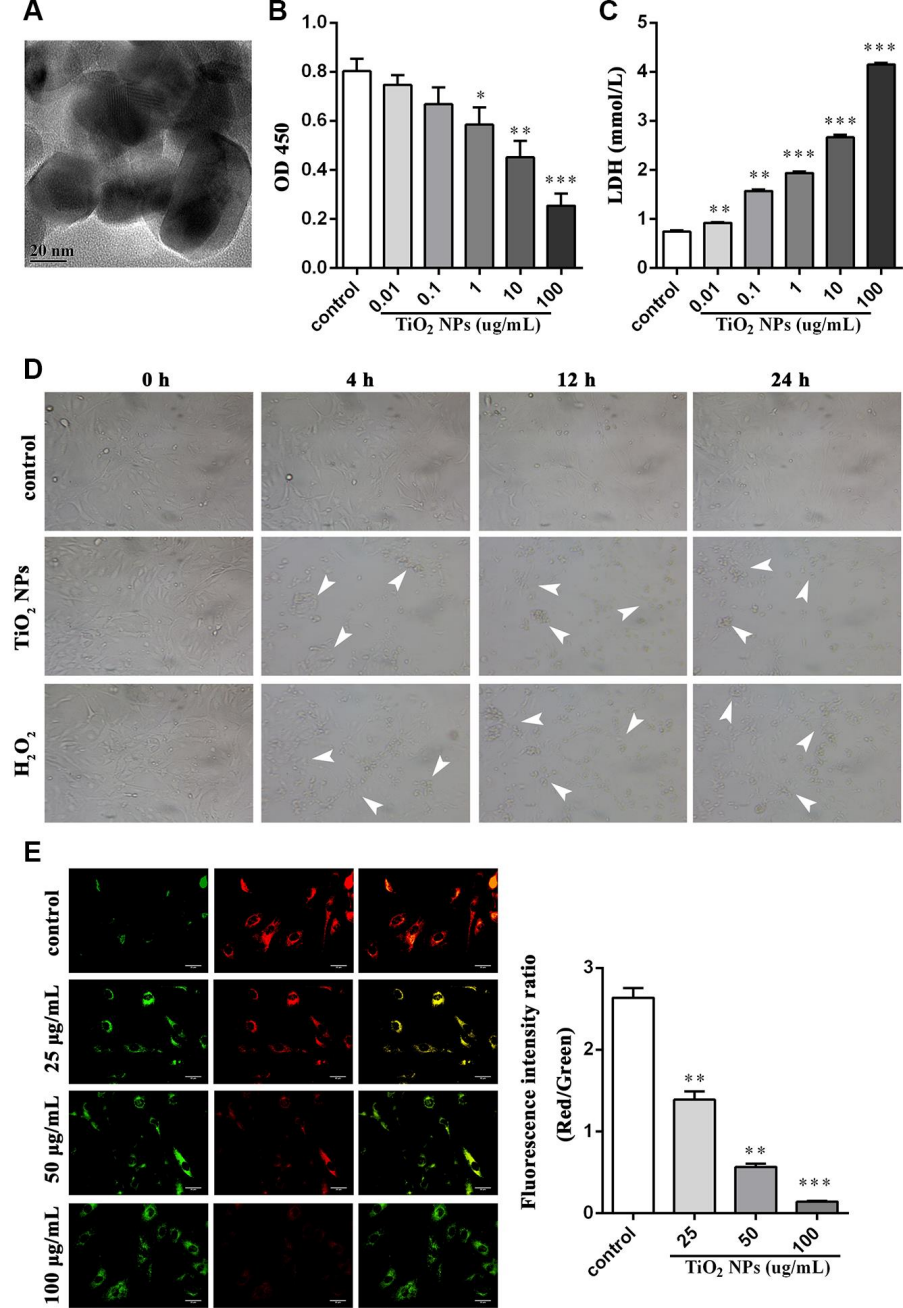
**TiO_2_ NPs boosted cell damage of corneal endothelial cells.** (**A**) Detection of shape and size of TiO_2_ NPs by TEM, bar = 20 nm. (**B**) Cell viability of TiO_2_ NPs-treated primary endothelial cells. (**C**) Measurement of LDH content in TiO_2_ NPs-treated primary endothelial cells. (**D**) Morphological analysis of TiO_2_ NPs-treated primary endothelial cells by inverted microscope, bar = 50 μm. (**E**) Mitochondrial membrane potential of TiO_2_ NPs-treated primary endothelial cells by using JC-1, bar = 30 μm. ^*^P<0.05, ^**^P<0.01, ^***^P<0.001.

First, TiO_2_ NPs were observed by using transmission electron microscope (Figure 1A). Next, we treated corneal endothelial cells with different concentration of TiO_2_ NPs for 24 h to examine cell viability. The results displayed that TiO_2_ NPs decreased cell viability in a concentration-dependent manner (Figure 1B). Moreover, LDH content was significantly increased by TiO_2_ NPs treatment (Figure 1C). The morphological analysis showed that the number of shrunken cells and dead cells was increased, and cell density was decreased when corneal endothelial cells were disposed to TiO_2_ NPs (Figure 1D). To better illustrate the oxidative damage of corneal endothelial cells, we took H_2_O_2_ treatment as the positive control (Figure 1C). In addition, we detected cell damage from a more microscopic point of view by evaluating mitochondrial membrane potential and found TiO_2_ NPs decreased red fluorescence intensity and increased green fluorescence intensity (Figure 1E), suggesting that TiO_2_ NPs led to mitochondrial damage.

### Oxidative damage of TiO_2_ NPs to corneal endothelial cells

**Figure 2:**
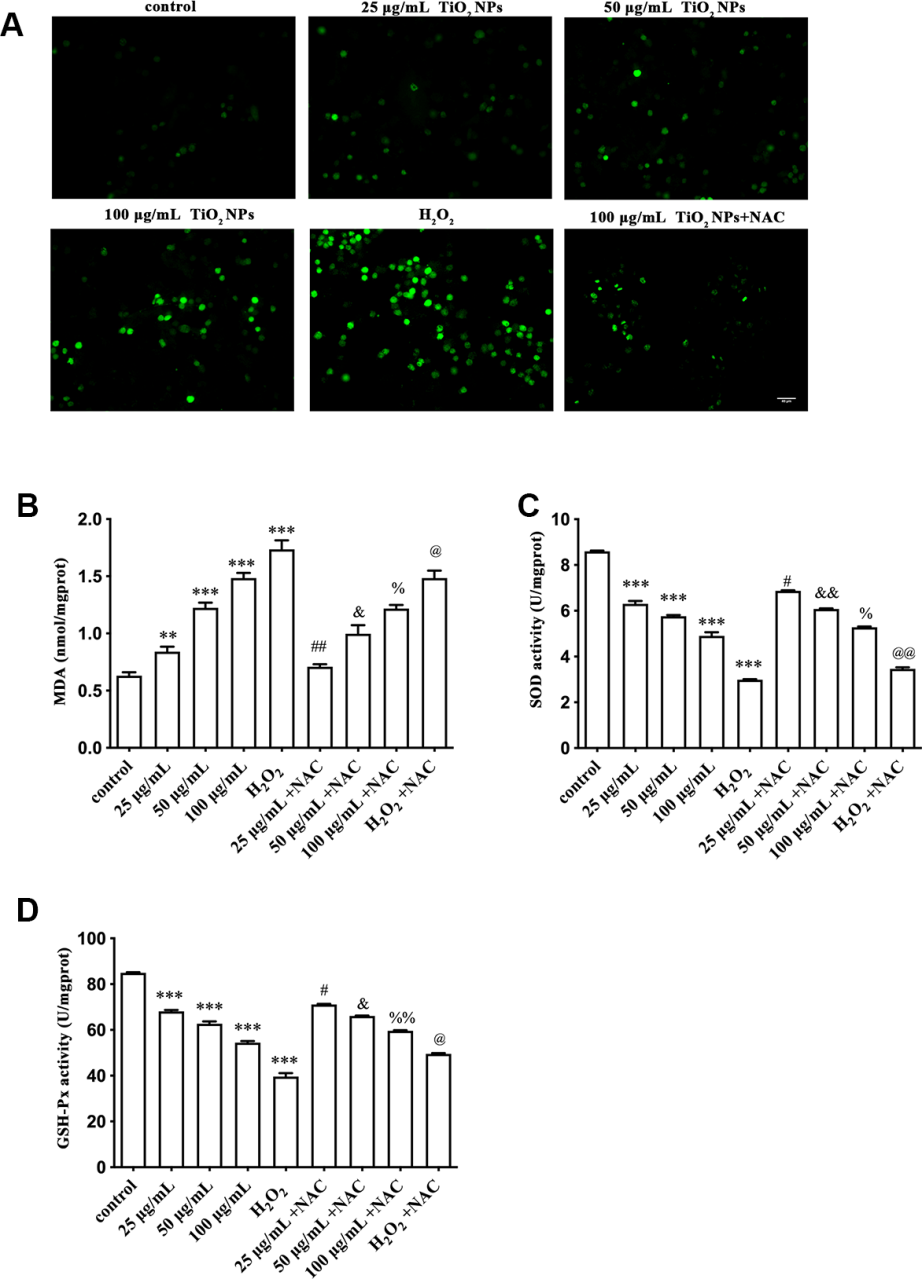
**Oxidative damage of TiO_2_ NPs to corneal endothelial cells.** (**A**) Intracellular ROS of corneal endothelial cells with indicated treatment, bar = 40 μm. Detection of MDA content (**B**), SOD activity (**C**) and GSH-Px activity (**D**) in primary corneal endothelial cells with indicated treatment. ^**^P<0.01 vs. control group, ^***^P<0.001 vs. control group, ^#^P<0.05 vs. 25 μg/mL group, ^##^P<0.01 vs. 25 μg/mL group, ^&^P<0.05 vs. 50 μg/mL group, ^&&^P<0.01 vs. 50 μg/mL group, ^%^P<0.05 vs. 100 μg/mL group, ^%%^P<0.01 vs. H_2_O_2_ group, ^@^P<0.05 vs. 50 μg/mL group, ^@@^P<0.01 vs. H_2_O_2_ group.

To further determine the oxidative damage of TiO_2_ NPs on corneal endothelial cells, we detected the following indicators and H_2_O_2_ treatment was used as the positive control. DCFH-DA is widely used to detect intracellular ROS [20]. We observed that TiO_2_ NPs augmented fluorescence intensity (Figure 2A), indicating the elevation of ROS generation. Meanwhile, NAC, a potent scavenger of ROS, could weaken the increased fluorescence intensity induced by TiO_2_ NPs (Figure 2A). In addition, TiO_2_ NPs increased MDA content in corneal endothelial cells, and NAC could antagonize the harmful role of TiO_2_ NPs (Figure 2B). We also found that the activity of either SOD or GSH-Px, two major enzymes acting as antioxidants, was decreased by TiO_2_ NPs in corneal endothelial cells, which was partially reversed by NAC treatment (Figure 2C and 2D).

### TiO_2_ NPs caused apoptosis and cell cycle arrest of corneal endothelial cells

**Figure 3:**
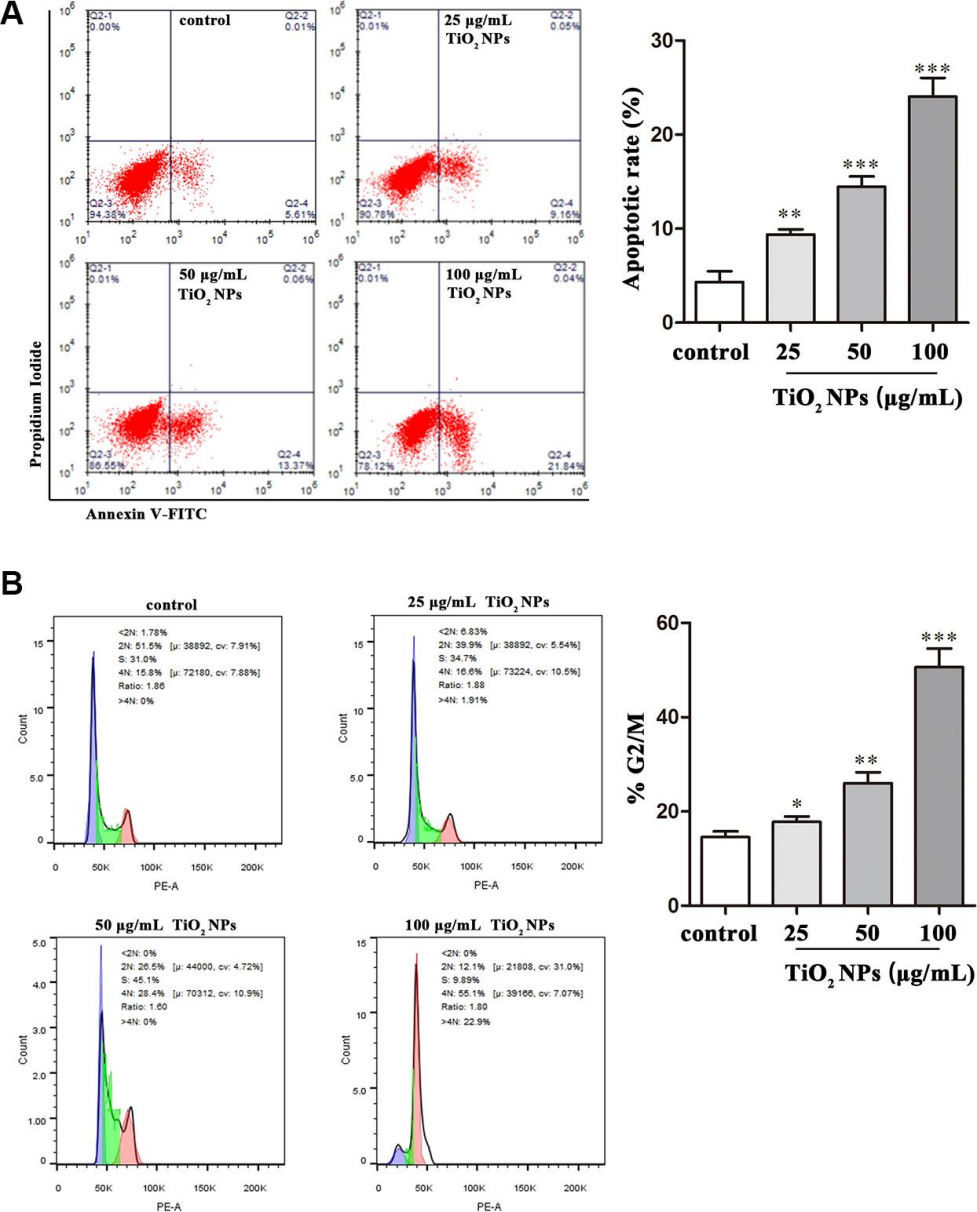
**TiO_2_ NPs caused apoptosis and cell cycle arrest of corneal endothelial cells.** Apoptosis analysis (**A**) and cell cycle analysis (**B**) of TiO_2_ NPs-treated primary corneal endothelial cells by using flow cytometry. ^*^P<0.05, ^**^P<0.01, ^***^P<0.001.

Apoptosis and cell cycle arrest are two biological indicators that inhibit cell proliferation. The flow cytometry analysis revealed that TiO_2_ NPs promoted apoptotic rate of corneal endothelial cells in a concentration-dependent manner (Figure 3A). Additionally, as was shown in Figure 3B, the proportion of cell cycle G2/M phase arrest was increased by TiO_2_ NPs treatment in a concentration-dependent manner (Figure 3B).

### TiO_2_ NPs activated Nrf2/ARE signaling pathway in corneal endothelial cells

**Figure 4:**
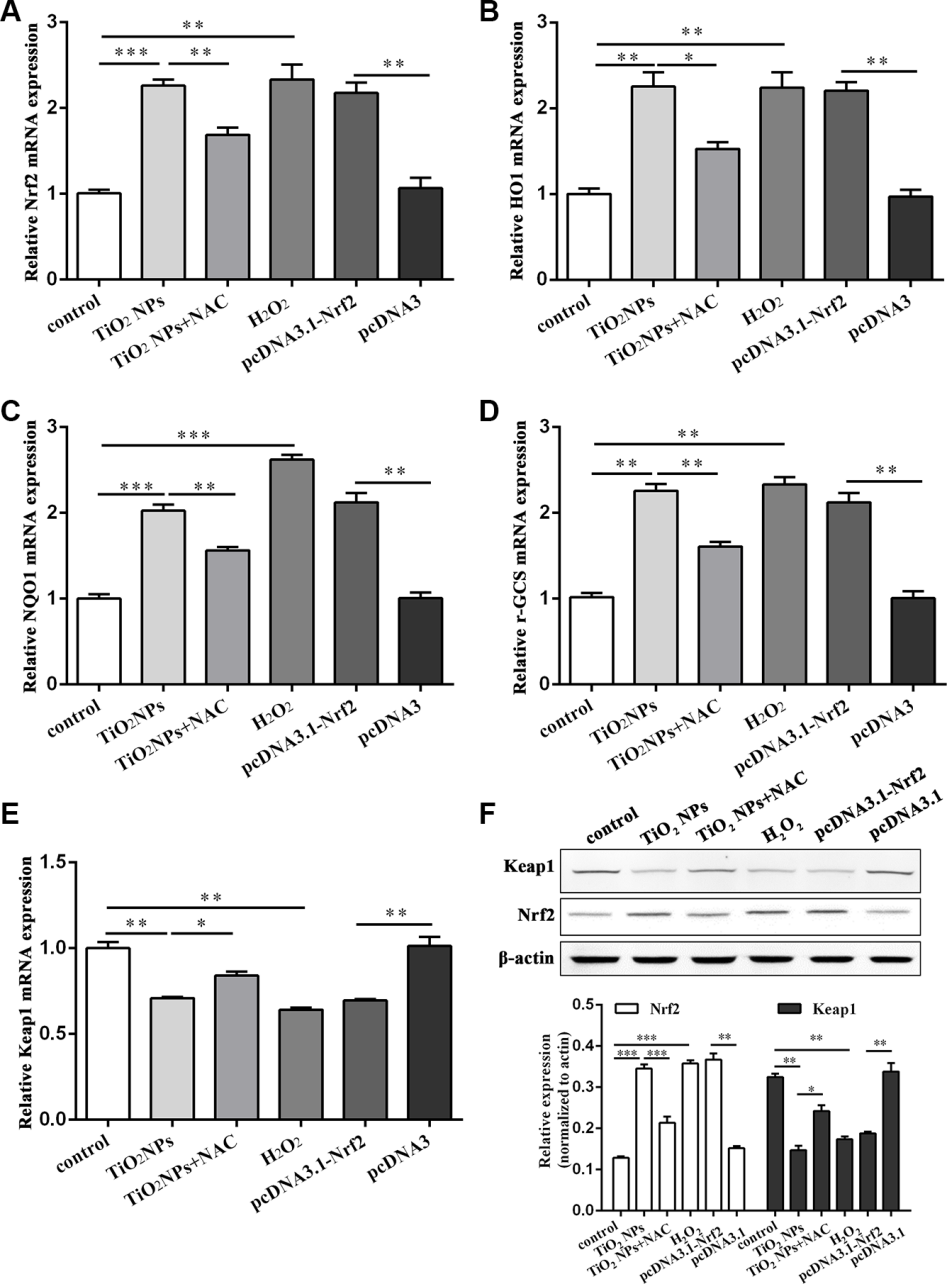
**TiO_2_ NPs activated Nrf2/ARE signaling pathway in corneal endothelial cells.** The mRNA level of Nrf2 (**A**), HO1 (**B**), NQO1 (**C**), γ-GCS (**D**), Keap1 (**E**) in primary corneal endothelial cells with indicated treatment. (**F**) Protein level of Keap1 and Nrf2 in primary corneal endothelial cells with indicated treatment. ^*^P<0.05, ^**^P<0.01, ^***^P<0.001.

Nrf2/ARE signaling pathway is a key component to exert an antioxidant role [21]. We found that TiO_2_ NPs treatment significantly upregulated the mRNA level of Nrf2 in corneal endothelial cells (Figure 4A). Additionally, Nrf2 overexpression plasmid was transfected into corneal endothelial cells as the positive control. Further experiments revealed that either TiO_2_ NPs treatment or Nrf2 overexpression could dramatically increase the mRNA level of Nrf2 downstream molecules HO1, NQO1 and γ-GCS (Figure 4B–4D). Conversely, the mRNA level of Keap1 was decreased by TiO_2_ NPs treatment and Nrf2 overexpression (Figure 4E). In addition, we found that NAC could partially antagonize TiO_2_ NPs-induced aberrant mRNA levels of Nrf2, HO1, NQO1, γ-GCS and Keap1 (Figure 4A–4E). Western blot analysis by detecting the protein levels of Nrf2 and Keap1 showed the similar results as RT-PCR did in corneal endothelial cells (Figure 4F). In parallel, H_2_O_2_ treatment displayed the similar results as TiO_2_ NPs did (Figure 4A–4F). These findings suggested that TiO_2_ NPs activated Nrf2-related signaling pathway in corneal endothelial cells.

### Regulation of functional proteins by TiO_2_ NPs in corneal endothelial cells

**Figure 5:**
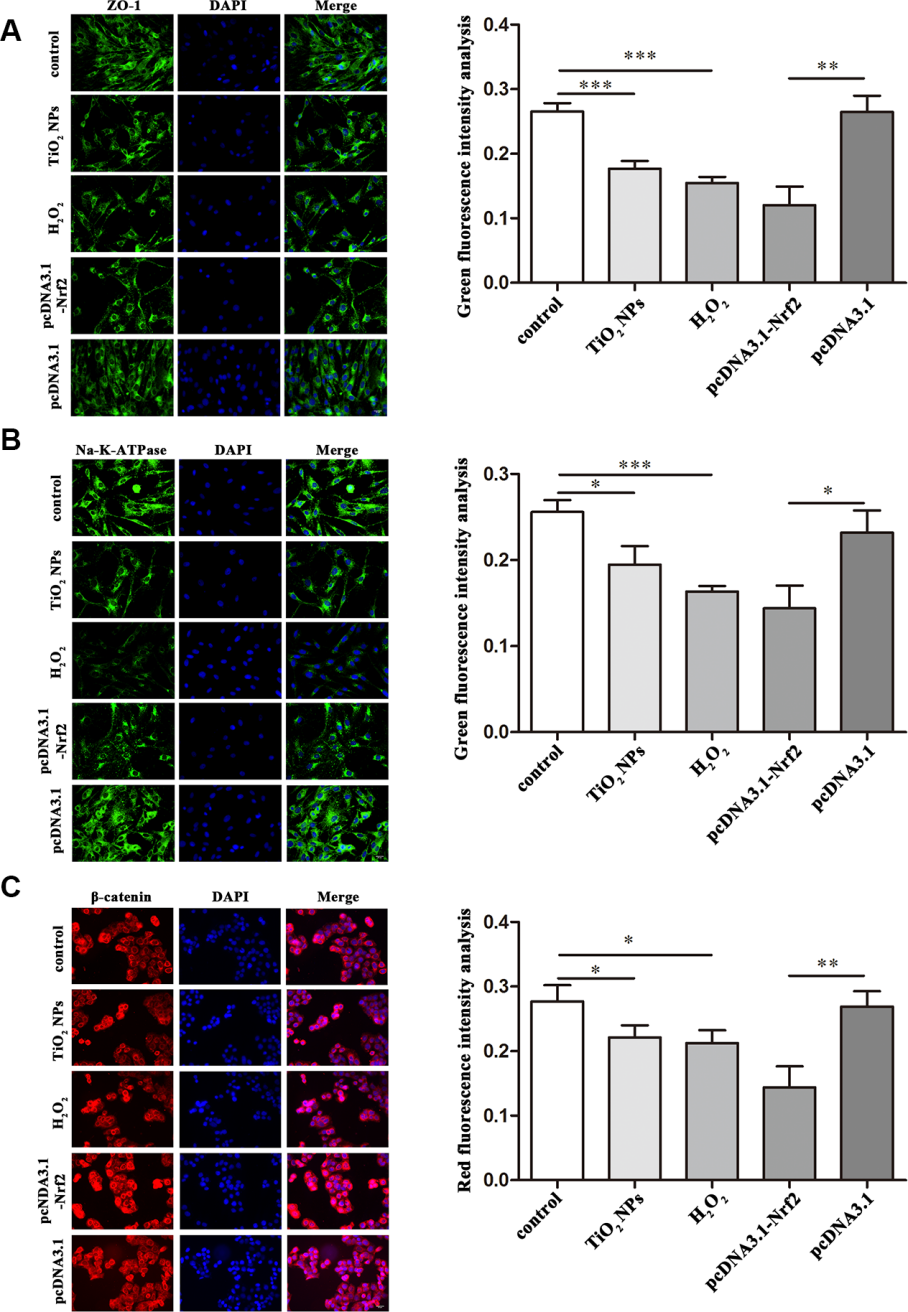
**Regulation of functional proteins by TiO_2_ NPs in corneal endothelial cells.** Immunofluorescence analysis of the expression of ZO-1 (**A**), Na-K-ATPase (**B**) and β-catenin (**C**) in primary corneal endothelial cells with TiO_2_ NPs treatment, H_2_O_2_ treatment or Nrf2 overexpression. bar = 20 μm. *P<0.05, **P<0.01, ***P<0.001.

Next, we investigated the role of TiO_2_ NPs in regulation of cell-cell contact-related proteins. The immunofluorescence analysis showed that TiO_2_ NPs treatment reduced the expression of ZO-1 and Na-K-ATPase in corneal endothelial cells (Figure 5A and 5B). Moreover, we found the expression of β-catenin in nucleus of corneal endothelial cells was significantly decreased by TiO_2_ NPs treatment (Figure 5C).

### TiO_2_ NPs damaged corneas *in vivo*

**Figure 6:**
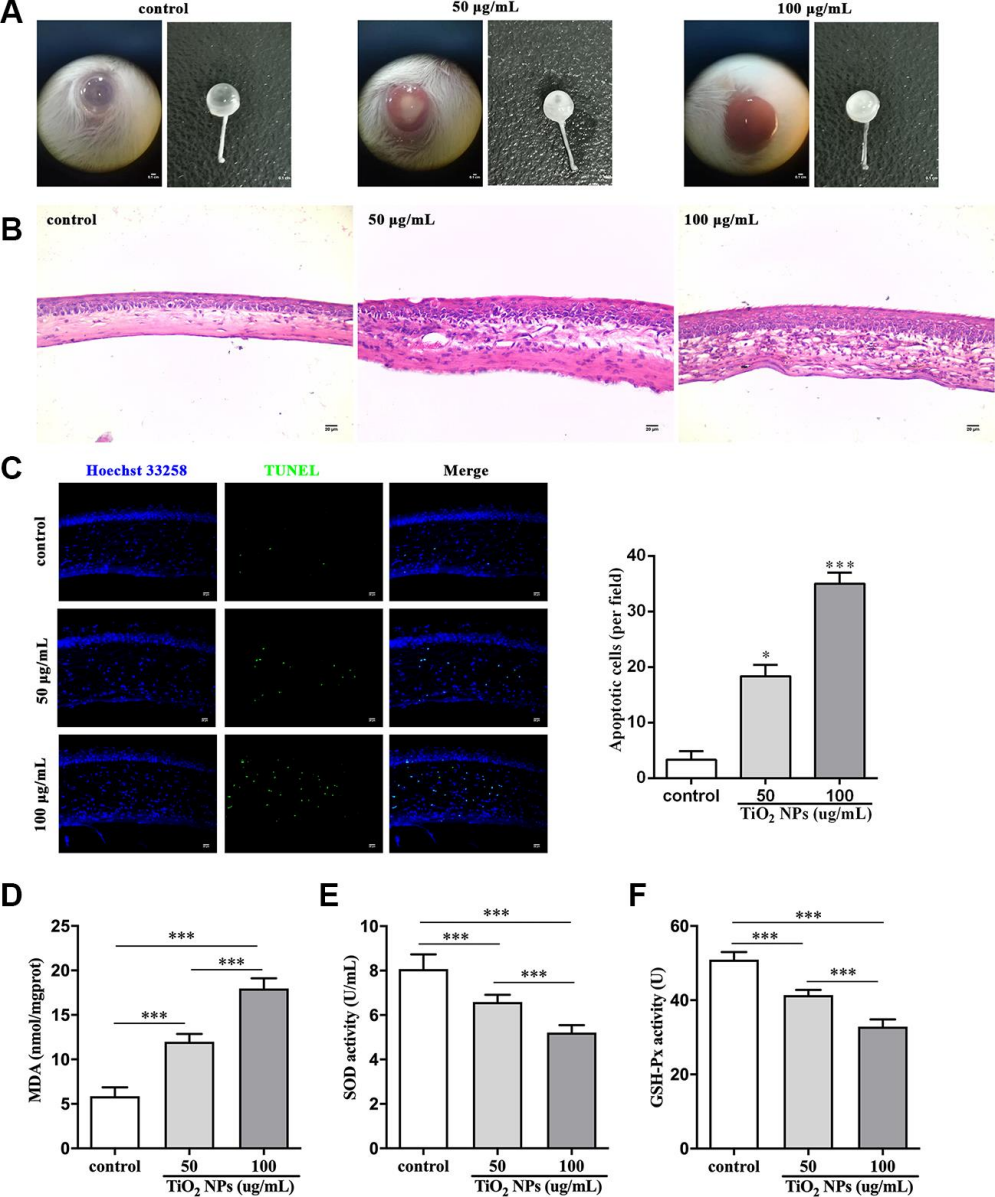
**TiO_2_ NPs damaged corneas *in vivo*.** (**A**) Images of eyeballs after indicated treatment *in vivo*, bar = 0.1 cm. (**B**) Histological observation of corneas with HE staining after TiO_2_ NPs treatment, bar = 20 μm. (**C**) Detection of apoptotic cells in cornea of TiO_2_ NPs-treated mice with TUNEL staining, bar = 20 μm. Measurement of MDA content (**D**), SOD activity (**E**) and GSH-Px activity (**F**) *in vivo*. *P<0.05, ***P<0.001.

Finally, we performed *in vivo* experiments to evaluate the harmful role of TiO_2_ NPs. After exposure to TiO_2_ NPs, the corneas displayed decreased transparency *in vivo* (Figure 6A). Histologically, the corneas were infiltrated with a large number of inflammatory cells (Figure 6B). The *in situ* TUNEL staining analysis showed that the number of TUNEL-positive cells was clearly increased in cornea of TiO_2_ NPs-treated mice (Figure 6C), indicating that TiO_2_ NPs promoted apoptosis of corneal cells. Moreover, MDA content was significantly increased and the activities of SOD and GSH-Px were remarkably decreased by TiO_2_ NPs in plasm of mice (Figure 6D-6F), which was in line with the results of *in vitro* assays (Figure 2B-2D). The *in vivo* experiments suggested that TiO_2_ NPs not only caused cornea damage, but also resulted in potentially systemic injury.

## DISCUSSION

TiO_2_ NPs, a kind of newly inorganic chemical material, have been widely used in a variety of fields involving chemistry, physics, biology and medicine. However, research on toxicity of TiO_2_ NPs has not kept pace with its application. In the present study, we found TiO_2_ NPs damaged morphology and cell viability of corneal endothelial cells. Moreover, biochemical indicators revealed that antioxidant NAC restrained TiO_2_ NPs-induce oxidative injury by evaluating ROS generation, MDA content, and the activities of antioxidases SOD and GSH-Px.

Currently, oxidative stress is the dominating factor that induces toxic effects of TiO_2_ NPs to organisms [17, 22, 23]. ROS are intermediates and end products during redox process, and ROS generation and elimination display a dynamic equilibrium state [24]. It has been reported that moderate ROS production is necessary to maintain normal physiological activities of cells and organisms [25]. Therefore, ROS overproduction could contribute to pathophysiological foundation of multiple diseases. MDA, a kind of degradation product formed in the process of lipid peroxidation, is a common indicator to reflect oxidative damage of cells [26]. We found that ROS generation in TiO_2_ NPs-treated corneal endothelial cells was accompanied by the increased MDA content. Moreover, TiO_2_ NPs restrained SOD activity and GSH-Px activity of corneal endothelial cells. SOD and GSH-Px are two major enzymes that play a crucial role in anti-oxidative damage [27]. *In vivo* experiments suggested that TiO_2_ NPs could reduce the activities of SOD and GSH-Px in cortex and hippocampus of mice [28]. Abbasi-Oshaghi et al reported that TiO_2_ NPs promoted activation of NLRP3 inflammation and ROS generation, and reduced expression and activities of a variety of antioxidases in liver [29]. In addition, we found that TiO_2_ NPs led to cell cycle G2/M phase arrest of primary corneal endothelial cells. It has been widely reported that ROS induces DNA damage by oxidized modification of bases and breaking DNA strands [30]. DNA damage contributes to cell cycle arrest to provide sufficient time for repairing, thus avoiding the damage to be inherited to daughter cells; when the damage could not be efficiently repaired, cell apoptosis occurs [31, 32]. Kansara et al demonstrated that TiO_2_ NPs-mediated upregulation of CdC2 and downregulation of Cyclin B1 were involved in cell cycle G2/M phase arrest and apoptosis of human alveolar cells [8]. We also found that TiO_2_ NPs-induced apoptosis of corneal endothelial cells was accompanied by the remarkably increased proportion of cell cycle G2/M phase.

Although the toxic effect of TiO_2_ NPs has been extensively investigated, the exact underlying mechanism of TiO_2_ NPs-related cell injury is still unclear. A recent research suggested that TGF-β1/Smad3/p38 signaling pathway participated in TiO_2_ NPs-induced renal cell damage [33]. In breast cancer cells, TiO_2_ NPs inactivated EGFR to inhibit activation of the downstream signaling pathway AKT and ERK, reducing cell adhesion and facilitating cell apoptosis [34]. In our study, we found Nrf2/ARE signaling pathway might contribute to the pathogenesis of TiO_2_ NPs-induced corneal endothelial cell injury. Normally, Nrf2 is bonded and inactivated by Keap1 in cytoplasm; when stimuli enable oxidative stress in cells, the negative regulation of Keap1 on Nrf2 is thus inhibited, and Nrf2 transfers to nucleus to interact with ARE so as to facilitate the expression of the downstream molecules [21]. Nrf2 is a key transcription factor that participates in anti-oxidative stress damage. Our findings indicated that TiO_2_ NPs promoted the expression of Nrf2 and target genes of Nrf2/ARE signaling pathway (HO1, NQO1 and γ-GCS). HO1, the inducible type of heme oxygenase, is a kind of rate-limiting enzyme that could degrade abnormal hemoglobin in blood [35]. NQO1 belongs to phase II enzymes, providing a protective role in oxidative damage. It has been verified that both HO1 and NQO1 are involved in corneal endothelial cell injury induced by physical and chemical factors [36, 37]. We further identified γ-GCS as a downstream molecule of Nrf2 in TiO_2_ NPs-induced oxidative damage of corneal endothelial cells.

Additionally, we found downregulation of ZO-1, Na-K-ATPase and β-catenin in TiO_2_ NPs-treated corneal endothelial cells. ZO-1 is tight junction protein that is involved in signal transduction at cell-cell junctions, which is also a key mediator for cell proliferation [38]. Na-K-ATPase is an enzyme that drives sodium and potassium to maintain cell homeostasis [39]. A recent study revealed that Roof plate-specific spondin 1-induced proliferation of corneal endothelial cells was associated with the two functional proteins ZO-1 and Na-K-ATPase, and nuclear translocation of β-catenin boosted the expression of proliferation-associated Cyclins [40]. Hong et al demonstrated that TiO_2_ NPs downregulated the protein level of Wnt3a and β-catenin, and inactivated Wnt3a/β-catenin signaling pathway to block development of neurons [41]. Combined with these results, our research suggested that downregulation of ZO-1, Na-K-ATPase and β-catenin might contribute to the decreased proliferation of TiO_2_ NPs-treated corneal endothelial cells. Taken together, our findings uncovered the crucial role of Nrf2/ARE signaling pathway and functional proteins ZO-1, Na-K-ATPase and β-catenin in TiO_2_ NPs-induced corneal endothelial cell injury, providing an alternative insight into the toxic effects of TiO_2_ NPs.

## MATERIALS AND METHODS

### Shape and size of TiO_2_ NPs

TiO_2_ NPs powder (Sigma-Aldrich, Germany) was heated at 120° C for 2 h and dissolved into sterile distilled water. After cooling naturally, the TiO_2_ NPs solution was stored at 4° C. In addition, the TiO_2_ NPs solution was vibrated by ultrasonic oscillation for 30 min before the assays. Observation of TiO_2_ NPs was detected by transmission electron microscope (TEM, Hitachi, Japan).

### Animal preparation

BALB/c mice (7-8-week-old) were purchased from Animal Laboratory Center of Weifang Medical University and raised in cages at 20° C ~ 24° C. The mice were free to obtain food and water and housed in the 12-hour light/dark cycle. The animal experiments were authorized by Animal Care and Use Committee of Weifang Medical University, and the procedures were act in accordance with the requests of Animal Care and Use Committee of Weifang Medical University. The mice were randomly divided into three groups: control group (n=15), 50 μg/mL TiO_2_ NPs group (n=15) and 100 μg/mL TiO_2_ NPs group (n=15). Eyes of mice in control group were exposed to distilled water and eyes of mice in the other two groups were exposed to different concentration of TiO_2_ NPs solution. All mice were treated once a day for one week, followed by being narcotized with 5% chloral hydrate to remove eyeballs.

### Hematoxylin-eosin (HE) staining

The procedure of HE staining for corneas was processed as previously shown [42] with a slight modification. In brief, the isolated tissues were fixed with 4% paraformaldehyde followed by dehydration and being stained by using Hematoxylin and Eosin Staining Kit (Beyotime, China). The images were observed and obtained with microscopy (Olympus, Japan).

### *In situ* TUNEL staining

The isolated corneas were incubated with TUNEL solution (Beyotime, China) to detect apoptosis *in situ*, and nuclei were stained with Hoechst 33285 (Beyotime, China). The detailed method was carried out in accordance with the manufacturer’s guidelines.

### Isolation of mouse primary corneal endothelial cells

BALB/c mice, aged 4-5 weeks, were obtained from the Animal Experimental Center of Weifang Medical University. The procedures of isolation of mouse corneal buttons were conducted as previously reported [43] with a modification. Briefly, eyes of mice were carefully removed after mice were killed, followed by being washed with phosphate-buffered saline (PBS). Corneal buttons were isolated and washed with PBS. The corneal endothelium was digested by 0.25% Trypsin-EDTA (Thermo Fisher Scientific, USA) at 37° C for 20 min. The corneal endothelial cells were harvested and cultured in M199 Medium (Thermo Fisher Scientific, USA) with 10% fetal bovine serum (Gibco, USA), followed by being subcultured at 1:3 split and cultured in Incubator (Thermo Fisher Scientific, USA) at 37° C with 5% CO2.

### Detection of cell viability

Cell viability was evaluated by using Cell Counting Kit-8 (CCK8, Beyotime, China) according to the manufacturer’s instruction. Cells were seeded in a 96-well plate at a density of 8000 cells per well. After 24 h, the cells were treated with different concentration of TiO_2_ NPs for 24 h. 110 μL fresh medium containing 10 μL reagents was replaced in each well at 37° C for 2 h. The value of optical density (OD) was detected by Microplate Reader (Bio-Rad, USA) at 450 nm.

### Cytotoxicity assay

The content of lactate dehydrogenase (LDH) was detected by using LDH Cytotoxicity Assay Kit (Beyotime, China) to evaluate the cytotoxicity of TiO_2_ NPs according to the manufacturer’s guidelines. In brief, the cells were seeded in a 96-well plate at a density of 8000 cells per well. After 24 h, the cells were treated with different concentration of TiO_2_ NPs for 24 h. After centrifuging, 120 μL supernatant in each well was transferred into a new 96-well plate followed by adding the reagents, and the OD value was detected by Microplate Reader (Bio-Rad, USA) at 490 nm.

### Morphological analysis of TiO_2_ NPs-treated corneal endothelial cells

The primary corneal endothelial cells were disposed to either 25 μg/mL TiO_2_ NPs or 1.0 mM H_2_O_2_ for indicated time, followed by being observed by inverted microscope (Olympus, Japan). The magnification was 40 × 10.

### Measurement of mitochondrial membrane potential

Mitochondrial membrane potential assay kit with JC-1 (Beyotime, China) was utilized to determine mitochondrial membrane potential of TiO_2_ NPs-treated corneal endothelial cells in accordance with the manufacturer’s instrument. JC-1 aggregates were represented as red fluorescence and JC-1 monomers were indicated as green fluorescence.

### ROS detection

The intracellular ROS was determined by using ROS Assay Kit (Beyotime, China) according to the manufacturer’s guidelines. The cells were seeded in a 6-well plate and treated with different concentration of TiO_2_ NPs in the presence of NAC (10 mM, Beyotime, China). Next, 1.5 mL diluted DCFH-DA was added into each well for 20 min. Cells were washed with serum-free medium for three times and observed by fluorescence microscope (Olympus, Japan). After that, the cells were harvested and analyzed by BeamCyte (China).

### Measurement of malondialdehyde (MDA) content, superoxide dismutase (SOD) activity and glutathione peroxidase (GSH-Px) activity

MDA content, SOD activity and GSH-Px activity in primary endothelial cells and plasma were detected with Malondialdehyde Assay Kit (JianchengBio, China), Superoxide Dismutase Assay Kit (JianchengBio, China) and Glutathione Peroxidase Assay Kit (JianchengBio, China) in accordance with the manufacturer’s guidelines.

### Measurement of apoptosis

Apoptotic cells were detected by using Annexin V-FITC/PI Apoptosis Detection Kit (Beyotime, China). Briefly, cells were seeded into a 6-well plate and treated with different concentration of TiO_2_ NPs for 24 h. Next, the cells were washed with PBS for three times and stained with 5 μL Annexin V-FITC for 5 min in the dark at room temperature, followed by being added with 10 μL Propidium Iodide (PI) for 15 min. Next, the samples were detected with BeamCyte (China) and analyzed with CytoSYS 1.0 software. Apoptotic cells were represented as the Annexin V (+)/PI (-) cells.

### Cell cycle analysis

Cell Cycle and Apoptosis Kit (Beyotime, China) was utilized to detect cell cycle. Briefly, cells were washed with pre-cooled PBS and fixed with 70% ethanol for 24 h overnight. After that, 0.2% Triton X-100 was added into the cell solution, followed by re-suspended with 100 μg/mL RAase A for 30 min. Next, 10 μL PI solution was added for staining at 37° C in the dark. BeamCyte (China) was used to analyze the results.

### Cell transfection

The Nrf2 overexpression plasmid pcDNA3.1-Nrf2 was synthesized from Sangon Biotech (China). Cell transfection was performed by using Lipofectamine 3000 (Invitrogen, USA) in accordance with the manufacturer’s guidelines.

### Quantitative real time-polymerase chain reaction (qRT-PCR)

RNA was extracted by using TRIzol (Ambion, Germany). cDNA was acquired from the extracted RNA, and qRT-PCR was carried out by using SYBR Premix Ex Taq II Kit (Takara, Japan), and the expression level was calculated by using 2^-ΔΔCT^ methods. The primers were synthesized from Sangon Biotech (China) and shown in Table 1.

**Table 1:** The sequences of primers.

**Gene**	**Sequence (5’-3’)**
Nrf2	Forward: TGGTTAAGATGGTCCACACGG
	Reverse: GCTGGAAGCTCGGTGTTAGT
HO1	Forward: CACAGATGGCGTCACTTCGT
	Reverse: GGGCGCTTTTGTCTGTACC
NQO1	Forward: TGGCCAATGGTTCACCTACC
	Reverse: TTCACCCGTCCTGTTTGGAT
γ-GCS	Forward: AGGCGATCTCTCTCCACTGT
	Reverse: ATGCCAGTCTCACCTTTCGG
Keap1	Forward: GTTGCCATCCGGAGAGTTGT
	Reverse: CAGTGTGTGGCCTGTGTGAC
GAPDH	Forward: TGAAATGTGCACGCACCAAG
	Reverse: GGGAAGCAGCATTCAGGTCT

### Western blot

RIPA (Beyotime, China) was used to lyse the cells, and protein concentration was detected with BCA Kit (Beyotime, China). The protein was separated by SDS-PAGE gels (Beyotime, China) and transferred to the PVDF membrane (Millipore, USA). After being blocked with 5% BSA, the membranes were incubated with primary antibodies (Rabbit polyclonal to Keap1, Abcam, USA; Rabbit monoclonal to Nrf2, Abcam, USA; Mouse monoclonal to beta Actin-Loading Control, Abcam, USA) overnight at 4° C. After being washed, the membranes were incubation with secondary antibodies (Beyotime, China). ChemDoc^TM^ XRS+ System (Bio-Rad, USA) was utilized to evaluate the protein bands.

### Immunofluorescence analysis

Primary corneal endothelial cells were fixed with 4% paraformaldehyde for 10 min. After being washed, cells were incubated with 0.1% Triton X-100 for 10 min at room temperature. Next, cells were washed and blocked with normal goat serum for 30 min followed by primary antibodies (Rabbit monoclonal to ZO-1 tight junction protein, Abcam, USA; Rabbit monoclonal to Sodium Potassium ATPase-Plasma Membrane Marker, Abcam, USA; Rabbit monoclonal to beta Catenin, Abcam, USA) overnight at 4° C. After being washed, the cells were incubated with secondary antibodies (Goat anti-rabbit IgG-Alexa Fluor 488 Conjugated, ZhuangzhiBIO, China; Goat anti-rabbit IgG-Cy3 Conjugated, ZhuangzhiBIO, China) for 1 h at room temperature in the dark, followed by being incubated with DAPI Staining Solution (Beyotime, China) for 10 min. Fluorescence analysis was performed with laser scanning confocal microscopy (Olympus, Japan).

### Statistical analysis

All the data in this work were represented as mean ± standard deviation (SD) with three independent experiments, and analyzed with non-parametric t tests by using the GraphPad Prism 5.0 Software (GraphPad, USA). P<0.05 was considered to be statistical significance.
